# Supplementary material for: Methanobrevibacter massiliense and Pyramidobacter piscolens Co-Culture Illustrates Transkingdom Symbiosis
Source: Microorganisms. 2024 Jan 20;12(1):215. doi: 10.3390/microorganisms12010215 (PMC10819710; doi:10.3390/microorganisms12010215)
Supplement: Supplementary file 1 [file microorganisms-12-00215-s001.zip › Supplementary data/Supplementary data S3 - S3.1-S3.2-S3.3.pdf]

Supplementary data S3

Figure S3.1: (a) Macroscopic observation of *M. massiliense*/*P. piscolens* Q8282 and Q8283 co-cultures, dark deposits are visible **(b)**. Macroscopic observation of *P. piscolens* Q8451 and Q8452 cultures, dark deposits are not visible. **(c)** Macroscopic observation of negative controls (GG medium inoculated with DPBS 1× water).

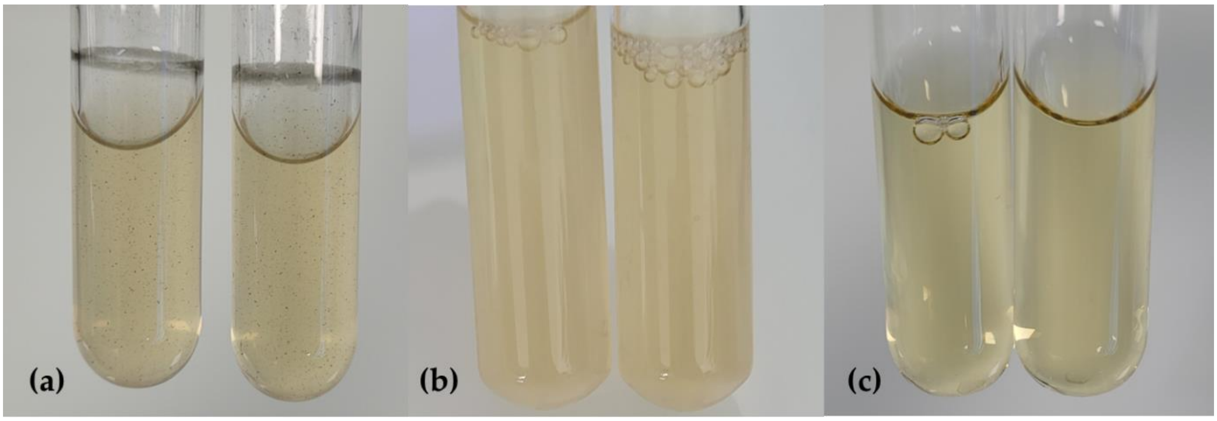

Figure S3.2 : Confocal microscopy of *M. massiliense*/ *P. piscolens* Q8282 **(line 1)**, of *P. piscolens* cultured in liquid GG medium **(line 2)** and *P. piscolens* cultured on COS medium **(line 3)**: brightfill mode view **(column 1)**: observation of diplo-coccobacilli, autofluorescence at 420 nm **(column 2)**: autofluorescent coccobacilli are visible for co-culture but no autofluorescent coccobacilli are visible for *P. piscolens* culture in GG medium or on COS medium, merged view of autofluorescence at 420 nm and brightfill mode **(column 3)**: autofluorescent coccobacilli are visible as well as no autofluorescent coccobacilli for co-culture but no autofluorescent coccobacilli are visible for *P. piscolens* culture in GG medium or on COS medium (LSM 900 (Carl Zeiss Microscopy GmbH)).

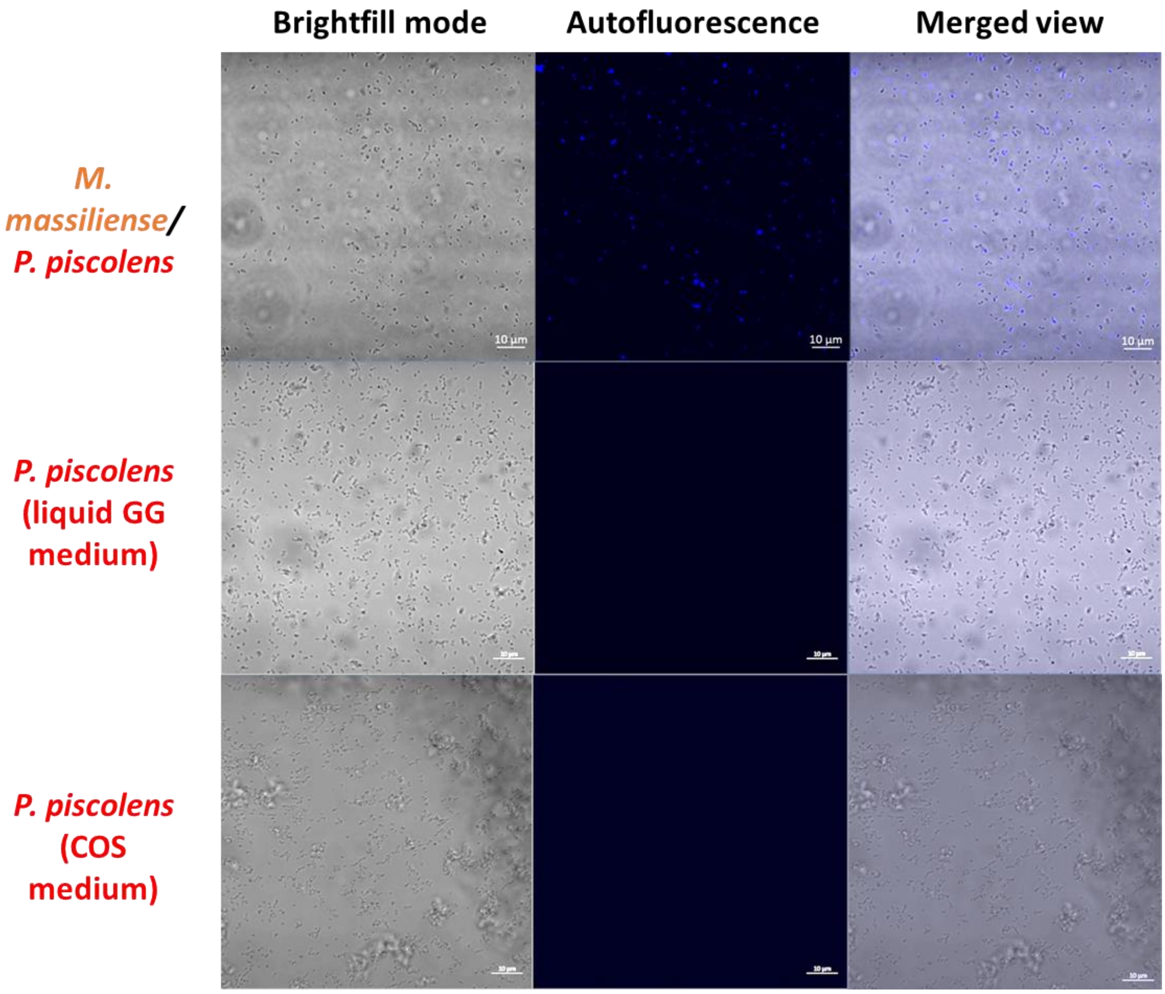

Figure S3.3 : Electron microscopy (TM4000 HITACHI, 10 KV, X 1.500). **(a)** Observation of *M. massiliense* and *P. piscolens* co-culture, coccobacilli are visible, but no morphologic features allow distinction between *M. massiliense* and *P. piscolens*. **(b)** and **(c)** Observation of deposits in *M. massiliense* and *P. piscolens* co-culture. **(d)** Observation of *P. piscolens* culture in GG medium. **(e)** Observation of *P. piscolens* culture in COS medium

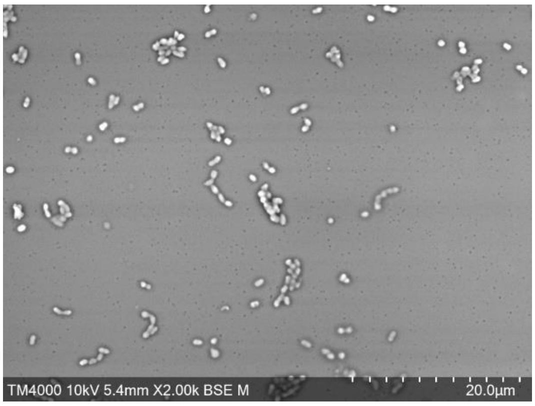

(a)

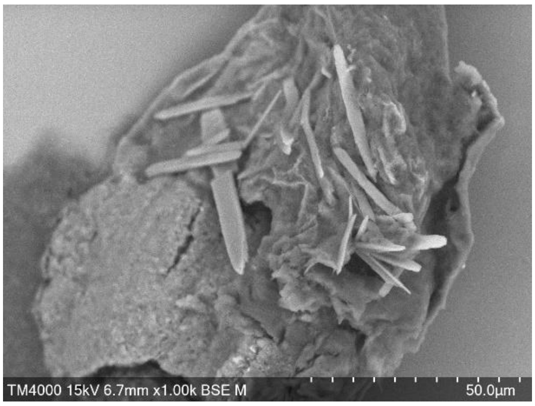

(b)

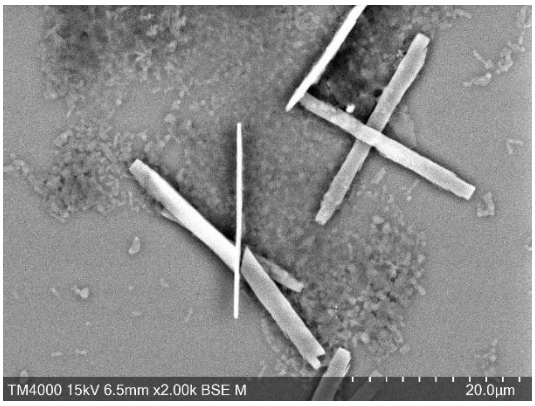

(c)

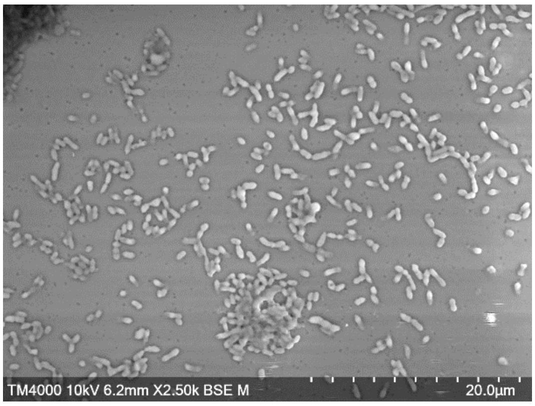

(d)

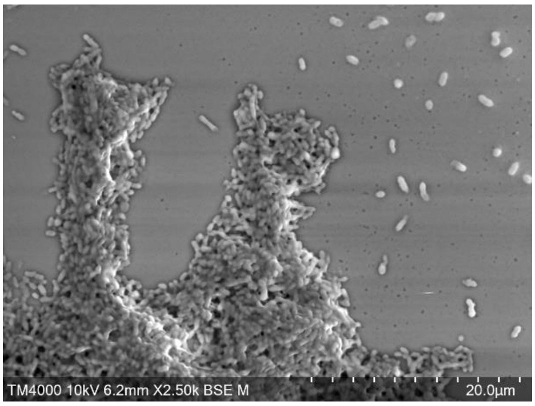

(e)
